# Draft genome sequences of two *Bacillaceae* strains isolated from Sua Pan, Makgadikgadi Basin, Botswana

**DOI:** 10.1128/mra.00976-26

**Published:** 2026-10-13

**Authors:** Daniel Loy, Lesedi Lebogang, Fulvio Franchi, Claire Batty, Karen Olsson-Francis, Michael C. Macey

**Affiliations:** 1 AstrobiologyOU, School of Environment, Earth and Ecosystem Sciences, The Open University, Milton Keynes, United Kingdom; 2 Department of Biological Sciences and Biotechnology, School of Life Sciences, Botswana International University of Science and Technology 357305, Palapye, Botswana; 3 Dipartimento di Scienze della Terrae Geoambientali, Università degli studi di Bari - Aldo Moro, Bari, Italy; 4 School of Geosciences, University of the Witwatersrand 37707, Johannesburg, South Africa; Wellesley College, Wellesley, Massachusetts, USA

**Keywords:** halophiles, spores, environmental microbiology

## Abstract

We report the draft genomes of halophilic *Sutcliffiella* and *Halalkalibacterium* strains isolated from hypersaline alkaline sediments of the Sua Pan salt flat in the Makgadikgadi Basin, Botswana. These sequences enable investigation of the biosynthetic potential and secondary metabolite systems of organisms from this extreme environment.

## ANNOUNCEMENT

Strains of *Bacillaceae* form endospores that protect against chemicals, drought, heat, and radiation, conferring stress tolerance (1, 2). To expand knowledge of *Bacillaceae* within the Makgadikgadi Basin (3–5), enrichments were established using sediment cores collected from Kubu Island (20°53′09.59″ S 25°49′59.88″ E–20°53′24.72″ S 25°49′51.59″ E), a granite outcrop in Sua Pan, on 19 January 2024. Sediment was incubated in 1:10 diluted Lysogeny Broth (LB; Invitrogen) or Tryptic Soy Broth (TSB; Oxoid) at 20°C for three months. Serial dilutions were plated onto solid LB or TSB and incubated at 30°C for 30 days. Single colonies were selected and purified through two successive rounds of streak plating, yielding *Sutcliffiella* sp. strain B65 and *Halalkalibacterium* sp. strain B6.

Biomass was harvested with sterile plastic loops from cultures grown on plates and was resuspended in DNA/RNA Shield (Zymo Research) and submitted to MicrobesNG (Birmingham). The cells were lysed using TE buffer containing lysozyme (MPBio), metapolyzyme (Sigma-Aldrich), and RNase A (ITW Reagents), followed by treatment with Proteinase K (VWR Chemicals, 0.1 mg/mL) and SDS (Sigma-Aldrich, 0.5% vol/vol). DNA was purified using Solid-Phase Reversible Immobilization beads, resuspended in 10 mM Tris-HCl (pH 8.0), and quantified with Quant-iT dsDNA HS assays (Thermo Fisher Scientific) using an Eppendorf AF2200 plate reader. DNA libraries were prepared using the Nextera XT library prep kit (Illumina, San Diego, USA), with a twofold increase in DNA input and a 45-s PCR extension step. Sequencing was performed on an Illumina NovaSeq 6000 using a 250-bp paired-end protocol.

Default settings were used throughout. Reads numbering 1,440,476 (B65) and 576,606 (B6) were quality-controlled using Trimmomatic (v0.30) (6), with adapter removal and quality trimming using a sliding-window quality threshold of Q15, generating 1,384,036 (B65) and 547,554 (B6) reads. Assemblies were generated using SPAdes (v3.7) (7) and assessed with CheckM (v1.4.0) (8). Coverage was calculated with BWA-MEM (0.7.17), SAMtools (v0.1.19), and BedTools genomecov (v2.2.7) (9–11). Taxonomic assignment was performed using Protologger2, incorporating FastANI-based average nucleotide identity comparisons against reference genomes (12, 13), and annotation was performed using the NCBI prokaryotic genome annotation pipeline (v6.10) (14).

*Sutcliffiella* sp. strain B65 shared 94% average nucleotide identity (ANI) with *Sutcliffiella cohnii*. The genome comprised 4.31 Mbp across 27 contigs, with 4,973 coding sequences, an N50 value of 653,609 bp, 35.86% GC content, and 146-fold coverage. *Halalkalibacterium* sp. strain B6 shared 95% ANI with *Halalkalibacterium halodurans*. The genome comprised 4.37 Mbp across 17 contigs, with an N50 value of 683,439 bp, 4,548 coding sequences, 43.24% GC content, and 63-fold coverage. CheckM estimated completeness and contamination values of 98.5% and 3.19% for strain B65 and 98.67% and 1.0% for strain B6, respectively.

antiSMASH (v8.2) (15) was run using default parameters and identified predicted biosynthetic gene clusters associated with terpene, polyketide, and peptide biosynthesis in strain B65 and clusters associated with nonribosomal peptides, lanthipeptides, β-lactones, arylpolyenes, siderophores, metallophores, autoinducers, and ectoine biosynthesis in strain B6. These findings highlight the biosynthetic potential of microorganisms inhabiting hypersaline environments (16–18).

## Data Availability

The Whole Genome Shotgun project has been deposited in DDBJ/ENA/GenBank under BioProject accession PRJNA1508428. Genome assemblies are available under accession numbers GCA_060301505.1 (*Sutcliffiella* sp. B65) and GCA_060301485.1 (*Halalkalibacterium* sp. B6). Associated BioSample accession numbers are SAMN62232802 and SAMN62232803. Raw sequence reads are available under accession numbers SRR40052210 and SRR40052211.
